# Ocular toxicity following carboplatin chemotherapy for neuroendocrine tumour of the bladder

**DOI:** 10.1177/10781552221122005

**Published:** 2022-08-24

**Authors:** Jia Ng, Muhayman Sadiq, Qasim Mansoor

**Affiliations:** 1 Ophthalmology Department, 111990 King’s College Hospital, London, UK; 2 King’s College London, 12196 Guy’s, King’s, and St Thomas’ School of Medicine, London, UK; 3 Ophthalmology Department, 156705 James Cook University Hospital, Middlesbrough, UK

**Keywords:** Carboplatin, ocular toxicity, chemotherapy, ophthalmology, bladder cancer

## Abstract

**Introduction:**

Carboplatin is a commonly used platinum analogue chemotherapeutic agent that is similar to cisplatin but is known to be better tolerated. This case report outlines a case of ocular toxicity following carboplatin chemotherapy used for the management of a neuroendocrine tumour of the bladder.

**Case report:**

A 70-year-old man with a history of neuroendocrine bladder cancer underwent chemotherapy with carboplatin and etoposide. He presented 4 weeks following his fourth chemotherapy cycle with a 1-week history of right eye blurriness. The patient had suffered a similar episode 2 weeks following his third chemotherapy cycle in his left eye. Carboplatin-induced ocular toxicity was suspected and his vision remained stable following cessation of carboplatin chemotherapy.

**Discussion:**

Current literature on carboplatin-induced ocular toxicity remains scanty, however, previous cases have reported symptoms beginning 5 days to 2 weeks following carboplatin use. Visual disturbance in the form of altered colour vision, blind spot, blurred vision and metamorphopsia have been reported by previous literature. This case report emphasised a case of bilateral sequential blurring of vision following carboplatin chemotherapy.

**Conclusion:**

It remains critical for ophthalmologists and oncologists to look out for ocular side effects of chemotherapy due to its devastating effects.

## Manuscript

A 70-year-old gentleman was referred to our ophthalmology unit with a 1-week history of blurred vision in the right eye (OD). He had a past medical history of invasive high-grade neuroendocrine bladder cancer, for which he underwent chemotherapy with carboplatin and etoposide. Upon presentation, it was 4 weeks following his fourth cycle of chemotherapy. On further history, he revealed that a similar episode occurred in his left eye (OS) 2 weeks following his third cycle of chemotherapy.

**Figure 1.**
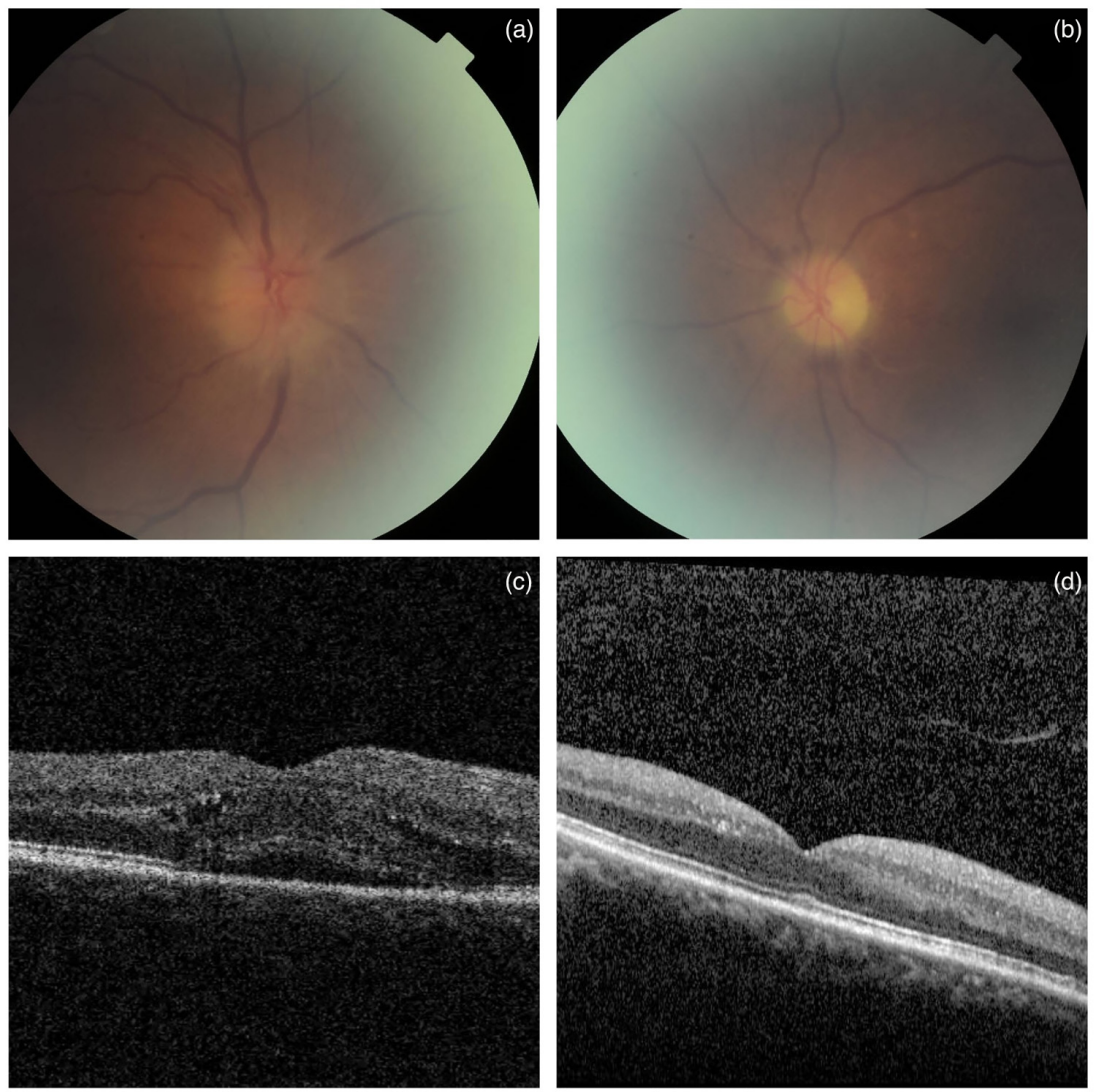
(a) Fundal examination showed segmental disc swelling superiorly associated with disc haemorrhage in OD. (b) Atrophic optic disc associated with sclerosed branch retinal artery was noted in the OS. (c and d) Optical coherence tomography showed subretinal fluid and intraretinal fluid in OD which resolved spontaneously 1 month later.

**Figure 2.**
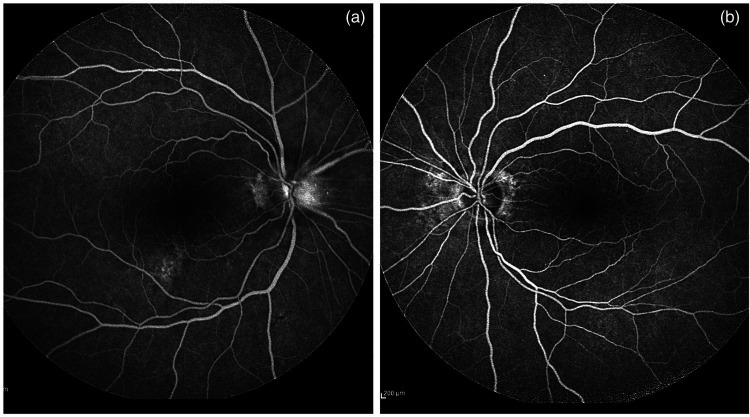
(a and b) Fundus fluorescein angiography showed bilateral late disc hyperfluorescence.

The best corrected visual acuity was 6/24 OD and count fingers OS with relative afferent pupillary defect OS. Colour vision on Ishihara plates was 13/13 OD and 0/13 OS. Fundal examination showed segmental disc swelling superiorly associated with disc haemorrhage OD (Figure 1(a)) and atrophic optic disc associated with sclerosed branch retinal artery OS (Figure 1(b)). Optical coherence tomography OD showed subretinal fluid and intraretinal fluid (Figure 1(c)) which resolved spontaneously 1 month later (Figure 1(d)). Fundus fluorescein angiography showed bilateral late disc hyperfluorescence (Figure 2(a) and (b)). Other cranial nerves examination was unremarkable.

**Figure 3.**
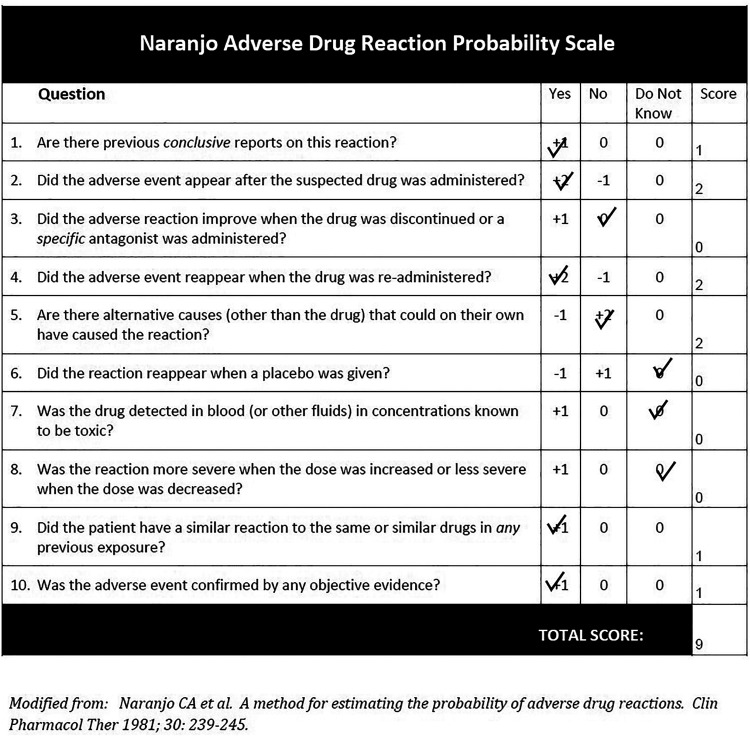
Calculation of the probability of an adverse drug reaction in the patient using the Naranjo Adverse Drug Reaction Probability Scale.

His blood pressure was 133/72 and HbA1c was 40mmol/mol. Laboratory test showed borderline cholesterol. His erythrocyte sedimentation rate and C-reactive protein were mildly elevated, a temporal artery biopsy was done and came back negative. CT brain showed no evidence of metastases and the patient was referred to a stroke physician where he was started on a prophylactic 75mg Aspirin dose. As he completed his fourth cycle of chemotherapy, he did not require any further cycles. His visual acuity 2 months following presentation remained stable. The patient's Naranjo Adverse Drug Reaction Score was calculated to be 9 (Figure 3).

## Discussion

Carboplatin is a commonly used platinum analogue chemotherapeutic agent that is similar to cisplatin but is known to be better tolerated. The pathogenesis of cisplatin/ carboplatin-induced ocular side effects is unclear. An in vitro study suggested cisplatin-induced platelet activation could explain thrombotic complications.^1^

Only a scanty number of cases have been reported on the ocular toxicity of carboplatin. Ocular symptoms described include pain, blurred vision, scattered blind spots, metamorphopsia and difficulty in reading.^2–5^ Visual symptoms typically present between 5 and 14 days following carboplatin administration. Clinical features described in published cases include unilateral or bilateral disc swelling associated with haemorrhages within the nerve fibre layer, pigmentary maculopathy, soft exudates, macular oedema, optic disc atrophy, uveal effusion glaucoma, exudative retinal detachment and ocular/orbital inflammation.^2–4^ In addition, abnormal colour vision, central scotoma on visual field testing and late optic disc hyperfluorescence have also been reported. More recently, carboplatin and paclitaxel combination has been reported to cause ischemic retinopathy presenting as cotton wool spots.^5^

It is apparent in our case that carboplatin-induced ocular toxicity can present in one eye and subsequently involve the other eye, leading to a bilateral permanent visual loss. It is important for oncologists and ophthalmologists to recognise this potentially devastating side effect and discontinue carboplatin in those patients with visual symptoms.
